# Do Elite and Amateur Soccer Players Outperform Non-Athletes on Neurocognitive Functioning? A Study Among 8-12 Year Old Children

**DOI:** 10.1371/journal.pone.0165741

**Published:** 2016-12-01

**Authors:** Lot Verburgh, Erik J. A. Scherder, Paul A. M. Van Lange, Jaap Oosterlaan

**Affiliations:** 1 Clinical Neuropsychology Section, Vrije Universiteit Amsterdam, Amsterdam, The Netherlands; 2 Dept. of Experimental and Applied Psychology, Vrije Universiteit Amsterdam, Amsterdam, The Netherlands; Universidad de Granada, SPAIN

## Abstract

**Aim:**

Research suggested a positive association between physical fitness and neurocognitive functioning in children. Aim of the present study is to investigate possible dose-response relationships between diverse daily physical activities and a broad range of neurocognitive functions in preadolescent children. Furthermore, the relationship between several sedentary behaviours, including TV-watching, gaming and computer time, and neurocognitive functioning will be investigated in this group of children.

**Methods:**

A total of 168 preadolescent boys, aged 8 to 12 years, were recruited from various locations, including primary schools, an amateur soccer club, and a professional soccer club, to increase variability in the amount of participation in sports. All children performed neurocognitive tasks measuring inhibition, short term memory, working memory, attention and information processing speed. Regression analyses examined the predictive power of a broad range of physical activities, including sports, active transport to school, physical education (PE), outdoor play, and sedentary behaviour such as TV-watching and gaming, for neurocognitive functioning.

**Results:**

Time spent in sports significantly accounted for the variance in inhibition, short term memory, working memory and lapses of attention, where more time spent in sports was associated with better performance. Outdoor play was also positively associated with working memory. In contrast, time spent on the computer was negatively associated with inhibition.

**Conclusions:**

Results of the current study suggest a positive relationship between participation in sports and several important neurocognitive functions. Interventions are recommended to increase sports participation and to reduce sedentary behaviour in preadolescent children.

## Introduction

Research has shown that 60 minutes moderate-to-vigorous physical activity (MVPA) per day is the minimum amount to benefit health in youth.[1] MVPA is defined as activity above three age-adjusted metabolic equivalents (METs), which are for example jogging, swimming or playing soccer.[2] Active involvement in physical exercise not only promotes physical health, but it also may enhance neurocognitive functioning (e.g. memory, information processing speed, inhibition). Yet most children and adolescents have not reached a healthy standard of exercise. According to research, 21% of the children up to 11 years of age, and 13% of the adolescents between 12 and 17 years of age, meet the recommended 60 minutes of daily moderate-to-vigorous physical activity (MVPA).[3]

Potential mechanisms underlying the beneficial effects of exercise on neurocognitive functioning include enhanced cerebral blood flow, growth factor release, neurogenesis, and angiogenesis.[4] Acute (immediate) beneficial effects of physical exercise in youth have been firmly established in a laboratory setting,[5] but it remains largely unknown whether structural daily life physical activities are also positively associated with neurocognitive functioning. Previous studies have shown that organized sports, active transport, physical education (PE) and outdoor play contribute significantly to the total MVPA in school-aged children, among which organized sports is most strongly associated with MVPA.[6–10] Therefore, the first aim of the present study is to investigate the association between a range of physical activities including sports, active transport, PE and outdoor play, and neurocognitive functioning (including short term memory, working memory, inhibition, attention and information processing speed) in preadolescent children. Such insights are not only of considerable scientific interest, but are also important for designing specific intervention programs to promote physical activity in youth. In this study we’ve focused on comparing three groups of preadolescent children varying in the amount of physical activity and sedentary behaviour: children who do not participate in any organized sports, children regularly participating in sports, and children very frequently participating in sports. For the latter two groups, we have chosen to include soccer players, as soccer is the most popular sports in the Netherlands, with a club member rate of 39% among boys between 5 and 18 years of age.[11]

Considering the above mentioned positive relationship between physical activity and neurocognitive functioning in young people, it is quite alarming that an increase in sedentary behaviour among youth has been demonstrated during the last decade.[12] Sedentary behaviour are activities such as TV-watching, playing computer games, and driving to school.[13] Sedentary behaviour is largely independent of physical activity, indicating that physical activities are not always replaced by sedentary behaviour.[14] A negative association has been suggested between sedentary behaviour and neurocognitive functioning.[4,15,16] The precise mechanisms are unknown, but sedentary behaviour may result in higher inflammation risks, disturbed insulin regulation, and high levels of triglycerides may play a role.[17] As far as we know, no study to date has investigated the relationship between sedentary behaviour and neurocognitive functioning in children.[4] Therefore, the second aim of the present study was to investigate the relationship between several sedentary behaviour including TV-watching, gaming and computer time, and neurocognitive functioning in preadolescent children.

## Methods

### Participants

A total of 168 preadolescent children, aged 8–12 years, were recruited from primary schools, an amateur soccer club, and a professional soccer club. The sample included 51 boys not involved in any organized sports (non-athletes), 48 boys who regularly participated in sports (non-elite soccer players), and 69 boys who participated very frequently in sports (elite soccer players). Non-athletes were not involved in any organized sports activities; they were neither member of a sports club, nor participating in an extracurricular sports program at school. Non-athletes were recruited at elementary schools. The non-elite players were recruited from an amateur soccer club. The elite players were recruited from the youth academy of a Dutch professional soccer club and were following the talent development program of the youth academy. Details on this group are provided in Verburgh et al.[18] Participants were free of known behavioural, learning and medical conditions that might impact neurocognitive functioning. To fully understand the tasks that were administered in this study, participants were included when they had an IQ>70 as measured by a short version of the Wechsler Intelligence Scale for Children III.[19] The study was approved by the Institutional Review Board (IRB) of the Vrije Universiteit Amsterdam (Faculty of Behavioural and Human Movement Sciences). All participants and parents and/or legal guardians were informed about the procedures of the study before giving their written informed consent prior to participation.

### Materials

#### Neurocognitive tasks

The Stop Signal task[18,20] was used to measure motor inhibition and involved two types of stimuli: Go stimuli and stop stimuli. Go stimuli were left- or right-pointing airplanes requiring a left or right button response, respectively. In a semi-randomly selected 25% of the trials, go stimuli were followed by a visual stop signal (traffic stop sign) superimposed on the go stimulus, requiring the participants to withhold their response. The delay between the go and stop signal (SSD) varied trial by trial using a tracking algorithm that increased or decreased the delay by 50 ms, depending upon whether or not the previous stop trial resulted in successful inhibition. This procedure yielded 50% successful inhibitions and 50% failed inhibitions. The dependent variable that reflects the latency of the inhibitory process is stop signal reaction time (SSRT). SSRT was calculated by subtracting average SSD from mean reaction time (MRT) calculated for correct responses on go trials. Shorter SSRTs reflect a faster and more efficient inhibitory process.

Two aspects of memory were assessed: short term memory and working memory.[21] Verbal short term memory was assessed using Digit Span Forwards of the Digit Span task of the WISC III.[19] Participants had to verbally reproduce dictated series of digits in a forward order, increasing in length after every two trials. Participants received one point for each correct response. Visuospatial short term memory was assessed using an adapted version of the task developed by Bergman-Nutley and colleagues,[22] in which participants were required to repeat sequences of yellow circles presented in a four by four grid (using the computer mouse) in a forward order (VSTM Forwards). Difficulty level was increased during the course of the task by manipulating position of the stimuli and increasing span. For both tasks, the total number of correct responses multiplied by highest difficulty level passed was calculated[23] and the composite score of the verbal short term memory and visuospatial short term memory task (the average of both z-scores) was included as dependent variable in the analyses.

Working memory was examined using the composite score (the average of both z-scores) of Digit Span Backwards of the Digit Span task of the WISC III[19] and the backward condition (VSTM Backwards) of the adapted version of the task developed by Bergman-Nutley and colleagues.[22] These conditions were similar to the forward conditions, but participants had to repeat the stimuli in reversed order, which appeals to working memory because information must be manipulated.[21]

Three aspects of attention were assessed: alerting, orienting, and executive attention. A modified version of the Attention Network Test (ANT)[24] was used to measure alerting and orienting attention and a modified version of the Flanker task was used to assess executive attention.[25] Alerting attention was measured by the relative change in MRT between alerting trials and neutral trials and orienting attention was measured by the relative change in MRT between orienting trials and alerting trials. The relative change in MRT between incongruent trials and congruent trials from the Flanker task was used as a measure of executive attention. For a detailed description of these measures, see Verburgh and colleagues[18].

Consistency in information processing speed was examined using individual response time distributions derived from correct go trials of the Stop Signal task. Each participant executed 145 go trials, which allows reliable analyses of processing speed.[26] The ex-Gaussian distribution model combines a normal distribution shape of individual reaction times with an exponential component on the right side of the distribution. With this model, Mu is calculated to determine the average speed of processing corrected for extreme slow responses. Furthermore, Sigma (fluctuations in speed of processing) and Tau (proportion of extreme slow responses, measuring lapses of attention) were calculated.[27]

### Estimated full-scale IQ

IQ was estimated by the Wechsler Intelligence Scale for Children III. Two subtests (Vocabulary and Block Design) were administered, correlating highly (*r* >.90) with full-scale IQ.[19]

### Body Mass Index

Participants’ height was measured using a stadiometer, with the child standing against a wall without shoes. Weight was measured for each participant to the nearest 0.1 kg by a weighing scale (Soehnle White Sense). Body Mass Index (BMI) was calculated from weight (in kg) / height × height in meters.[28]

### Physical activity and sedentary behaviour

Involvement in physical activities and sedentary behaviour were assessed using a questionnaire consisting of 13 questions on physical activity (e.g., ‘How many days a week are you going to school walking or cycling?’) and six on sedentary behaviour (e.g., ‘How many days a week do you watch television?’). Participants were required to indicate how many days per week and how many minutes per day they participated in each of the activities listed. Included dependent variables were total minutes spent in: Organized sports, active transport, PE, outdoor play, TV-watching, computer use, as well as active gaming (e.g., Wii Sports). Adequate reliability and validity have been reported for this questionnaire.[29]

### Procedure

Data of the non-athletes were collected at elementary schools during regular school hours or immediately after school. Data of the elite soccer players and non-elite soccer players were collected at the soccer club during the competitive soccer season. Participants from both soccer player groups were tested prior to soccer training, the non-athletes were tested on a day without PE class. All participants were tested in a quiet room by trained assessors using standardized instructions. There were two sessions with a duration of approximately one hour for each individual participant. First, body height and body weight were measured, followed by the WISC III and the neurocognitive tasks in fixed order.

### Statistical analyses

MATLAB was used to subtract Mu, Sigma, and Tau from the individual reaction times of the Stop Signal task. SPSS version 22.0 was used for all statistical analyses (SPSS IBM, New York, U.S.A). Five participants were removed from all analyses due to not attending the second test session, technical difficulties or not speaking fluent Dutch (N = 3 non-elite soccer players, N = 2 elite soccer player). For the 163 remaining cases, total missing data of demographic variables was less than 5% (N = 6 for IQ, N = 8 for the questionnaire on physical activity and sedentary behaviour in elite soccer players). Missing data were missing completely at random and were replaced by expectation maximization.[30] Standardized scores were used in all analyses and for the VSTM backwards and Digit Span Backwards, van der Waerden transformations were applied[30] as they were not normally distributed. Exploratory analyses on the nine neurocognitive measures showed only significant (and if so, small) correlations between some of the neurocognitive measures. This showed that each of the neurocognitive measures assesses unique and largely independent aspects of neurocognitive functioning. Possible group differences in physical activity, sedentary behaviour, age, BMI and IQ were tested using univariate analyses of variance (ANOVA) and were further explored with Tukey post hoc comparisons. Pearson correlations were performed to determine the possible relationship between the neurocognitive measures and age, BMI and IQ. If necessary, age, BMI and IQ were entered as a covariate in subsequent analyses. Next, following Field[31] a series of multiple backward regression analyses were conducted to investigate the relationship between the measures of physical activity and sedentary behaviour: organized sports, active transport, PE, outdoor play, TV-watching, computer use, as well as active gaming, and the neurocognitive measures: inhibition (SSRT), short term memory (Digit Span Forwards, VSWM Forwards), working memory (Digit Span Backwards, VSWM Backwards), attention (Alerting, Orienting, Executive Networks), and information processing speed (Mu, Sigma, Tau). The alpha-level was Bonferroni-adjusted for the number of predictors (α = .05/7), resulting in p-values smaller than 0.007 (two-tailed) considered statistically significant. Results were expressed in terms of R-squared (R^2^) and standardized regression coefficients (β) with values of 0.10, 0.30 and 0.50, referring to small, medium and large effects, respectively.[32]

## Results

### Preliminary analyses

Group characteristics and data on all neurocognitive measures are shown in Tables 1 and 2. Total minutes of physical activity and sedentary behaviour per week were not significantly correlated (*r* =. -08, *p* = .31). Age was associated with all assessed neurocognitive functions (0.26 > r’s < 0.53, .001 > p’s < .01) and was therefore included as covariate in all subsequent analyses. There were no significant relationships between both BMI and IQ and the neurocognitive measures, with two exceptions: IQ significantly correlated with both short term memory (r = .30, p < .001) and working memory (r = .32, p < .001). Because group differences were found for IQ (Table 2), analyses were performed with and without IQ as covariate in regression analyses with short term memory and working memory as dependent variables.

**Table 1:** Group characteristics.

		Non-Athletes (NA, N = 51)	Non-Elite Soccer Players (AP, N = 48)	Elite Soccer Players (EP, N = 69)	Test Statistic	p-value	Post hoc Tukey tests (*p* <.05)
		Mean (SD)	Mean (SD)	Mean (SD)			
**Age**		10.4 (1.2)	10.5 (1.3)	10.6 (1.4)	*F*(2,165) = .23	*p* = .79	
**Handedness (% right handed)**		84.1	81.6	88.4	χ^2^ = 1.1	*p* = .58	
**IQ**		102.3 (13.6)	101.6 (15.6)	93.6 (10.0)	*F*(2,165) = 8.6	*p* < .001	EP<NES = NA
**BMI**		17.8 (3.2)	17.2 (2.6)	17.8 (.78)	*F*(2,165) = .1.3	*p* = .18	
	Underweight (%)	4.5	12.2				
	Healthy (%)	75	77.6	100%			
	Overweight (%)	20.5	10.2				
**Physical activity**							
	Transport (min/week)^a^	96.8 (670)	111.2 (88.6)	126.3 (92.6)	*F*(2,165) = 1.7	*p* = .18	
	Physical education (min/week)	95.1 (30.3)	86.5 (39.8)	88.7 (57.8)	*F*(2,165) = .43	*p* = .65	
	Outdoor play (min/week)	588.4 (381)	813.2 (392)	470.9 (298.1)	*F*(2,165) = 13.7	*p* <.001	NES>NA
	Sports (min/week)	0	253.5 (100)	391 (49.1)	*F*(2,165) = 524	*p* <.001	EP>NES>NA
**Sedentary behavior**							
	TV watching (min/week)	587.3 (402.8)	484.2 (362)	349.2 (229.8)	*F*(2,165) = 7.5	*p* < .001	EP<NES = NA
	Computer/internet/gaming^b^ (min/week)	416.2 (480.1)	375.1 (270.8)	450.8 (450.8)	*F*(2,165) = 0.8	*p* = .45	
	Active gaming^c^ (min/week)	58.9 (109.9)	80.1 (131.8)	77.9 (98.1)	*F*(2,165) = .49	*p* = .61	

Note: NA = non-athletes; NES = non-elite soccer players; EP = elite soccer players; IQ = intelligent quotient; BMI = body mass index

^a^Actively walking or cycling to school.

^b^Using computer, internet, playing games or at game console (e.g. PlayStation, Nintendo).

^c^Playing active games on the computer or game console (e.g. Wii Sports).

**Table 2:** Group differences on neurocognitive measures.

	Non-Athletes (NA, N = 51)	Non-Elite Soccer Players (AP, N = 48)	Elite Soccer Players (EP, N = 69)	Test Statistic	p-value	Partial Eta^2^	Post hoc Tukey tests (*p* <.05)
	Mean (SD)	Mean (SD)	Mean (SD)				
**Motor inhibition**							
**SSRT**	270.8 (70.5)	280.6 (65.9)	227.6 (41.5)	*F*(2, 165) = 12.5	*p* < .001	.14	EP>NES = NA
**Short term memory**				*F*(2,165) = 4.4	*p =* .014	.05	EP >NA
**Verbal**	39.6 (14.1)	44.7 (18.2)	46.7 (16.9)				
**Visuospatial**	66.2 (27.5)	69.3 (30.5)	75.9 (23.3)				
**Working memory**				*F*(2,165) = 6.1	*p =* .002	.07	EP = NES>NA
**Verbal**	17.0 (8.9)	27.3 (16.2)	25.4 (25.4)				
**Visuospatial**	52.9 (28.3)	58.1 (34.4)	60.4 (28.4)				
**Attentional Networks**							
**Alerting**	26.5 (5.9)	27.1 (6.8)	26.2 (7.5)	*F*(2,165) = .67	*p =* .51		
**Orienting**	4.8 (7.9)	6.0 (7.9)	6.8 (7.1)	*F*(2,165) = 1.6	*p =* .20		
**Executive**	12.3 (10.6)	14.6 (8.3)	15.7 (6.7)	*F*(2,165) = 2.4	*p =* .10		
**Processing Speed**							
**Mu**	423.8 (68.1)	395.5 (57.9)	429.8 (103.2)	*F*(2,165) = 2.5	*p =* .16	.02	
**Sigma**	69.9 (30.6)	60.1 (19.8)	78.7 (44.6)	*F*(2,165) = 3.9	*p =* .25	.02	
**Tau**	135.3 (44)	112 (34.5)	98.5 (42.4)	*F*(2,165) = 11.1	*p* < .001	.13	EP = NES>NA

Note: NA = non-athletes; NES = non-elite soccer players; EP = elite soccer players; SSRT = stop signal reaction time. See main text for an explanation on how the measures were calculated.

### Regression analyses

Collinearity statistics of the predictors yielded tolerance values between 0.72 and 0.95, with variance inflation factors between 1.1 and 1.9, indicating that the validity of the regression models was not threatened by multicollinearity.

The multiple (backward) regression analyses revealed that time spent *in organized sports* was positively associated with inhibition (β = 0.25, p = .003, 95% CI 0.09–0.42, R^2^ = .084), short term memory (β = 0.18, p = .006, 95% CI 0.05–0.31, R^2^ = .082), working memory (β = 0.25, p < .001, 95% CI 0.12–0.38, R^2^ = .10), and lapses of attention (tau) (β = 0.32, p < .001, 95% CI 0.15–0.50, R^2^ = .11). Additional analyses with IQ as covariate for short term memory and working memory revealed the same significant results. The only difference was that for short term memory the Beta and effect size became larger, which could be explained by the somewhat lower IQ of the elite soccer player group, while the elite soccer players showed the best short term-memory scores (Table 2). As we found elite soccer players to outperform both the non-elite soccer players and the non-athletes on inhibition (Table 2) and the high amount of sports the elite players participated in, we reran the regression analysis with SSRT as dependent variable while eliminating the group of elite soccer players. This analysis allows us to examine the possibility that the found relationship between organized sports and inhibition was due to superior performance of the elite soccer players. Results showed that time spent in organized sports was not associated with inhibition (β = -0.16, p = .38, CI. -0.52–0.19) when the elite soccer players were excluded, indicating that the results of the regression analysis with all participants were largely driven by the scores on SSRT of the elite soccer players. This interpretation was further supported by the non-significant Pearson correlations between time spent in sports and inhibition within the non-elite and non-athlete group (r = .03, p = .78) and within the elite soccer player group (r = .07, p = .60). As was also reported in Table 2, there were group differences on short term memory, working memory and lapses of attention. Therefore, we repeated the method described above for inhibition (SSRT) for those three measures. By excluding the elite soccer players, sports was still associated with better performance on short term memory (β = 0.09, p = .02, 95% CI 0.014–0.17, R^2^ = .05) and working memory (β = 0.39, p < .001, 95% CI 0.009–0.25, R^2^ = .15). Sports was not associated with lapses of attention when the elite soccer players were excluded (β = 0.08, p = .15, 95% CI -0.003–0.018).

*Outdoor play* was positively associated with working memory in the analyses with and without IQ as covariate (β = 0.19, p = .002, 95% CI 0.07–0.30, R^2^ = .028, and β = 0.37, p = < .001, 95% CI 0.24–0.49, R^2^ = .10, respectively).

Last, regarding sedentary behaviour, *computer use* was negatively related to inhibition (β = -0.27, p = .001, 95% CI -0.42–-0.12, R^2^ = .024). The other measures of physical activity (PE and active transport) and sedentary behaviour (TV-watching and active gaming) were not associated with scores on any of the measured neurocognitive functions.

## Discussion

The present study addressed the relationship between both physical activity and sedentary behaviour and neurocognitive functioning in preadolescent boys. Results showed that time spent in organized sports was positively associated with short term memory, working memory, and lapses of attention. Moreover, time spent playing outdoors was also associated with working memory. In contrast, time spent at the computer or gaming was negatively associated with inhibition.

### Benefits of physical activity

The observed positive relationships between organized sports and the neurocognitive measures: short term memory, working memory, and lapses of attention, as well as the positive relationship between outdoor play and working memory are highly important, as these neurocognitive functions are the key to daily life functioning, including academic achievement, autonomous behaviour, and quality of life.[33] For example, working memory is a major mediator in academic achievement: It has been shown that working memory is highly predictive for reading and spelling achievement in school-aged children.[34] Our findings receive support from previous studies that observed enhanced working memory after single bouts[35] and regular sessions of physical exercise[36] in healthy preadolescent children, suggesting that even short sessions of active outdoor play during school time may be beneficial for working memory. Concerning lapses of attention (Tau), it has been shown that short losses of attention may lead to educational problems.[37] Interestingly, integrity in several important white matter tracts is positively associated with Tau.[38,39] Moreover, there is some evidence showing that physical exercise may lead to improved white matter integrity in these brain areas.[40,41]

Our findings raise the intriguing possibility that physical activity might be a promising method to enhance short term memory and working memory. When we excluded the group of elite soccer players from the analyses because they showed better performance on the inhibition, short term memory, working memory and attentional tasks, there still was a significant positive relationship between short term memory and working memory, and participation in soccer. In contrast, a recent study [42] showed that soccer players outperformed non-athletic children on a psychomotor vigilance task (PVT) and had better cardiovascular fitness. Interestingly, no relationship between cardiovascular fitness and the PVT was found, which may be due to the possibility of the PVT appealing less to executive functions, or methodological limitations (i.e. small sample size) of the study. Another possible explanation of the findings of Ballester and colleagues is the ‘cognitive component skills theory’ suggesting innate excellent cognitive skills in elite athletes. Indeed, several studies[18, 43, 44] show already differences between elite and sub-elite soccer players at a very young age. However it is still topic of debate whether the excellent cognitive skills of elite youth athletes result from training (e.g. a result of many training hours, high quality training facilities and coaches) or are innate. Our study adds to this debate by showing that especially on motor inhibition and lapses of attention, the elite soccer players showed superior performance. All in all, we believe that the current study together with the findings of our meta-analysis and other recent studies[5, 35, 36, 45], provides enough support for the recommendation to include more sports in the school curriculum, for instance by intensifying PE classes and by encouraging active play during recess at school.

Nevertheless, we emphasize the need for further research on the relationship between sports and neurocognitive functioning and moderators such as MVPA and, cardiovascular fitness. Other possible moderating factors such as improved motor skills[46] (needed in many neurocognitive tasks) or motivational aspects, may play a role as well in the relationship between sports participation and neurocognitive functioning.

### Costs of sedentary behaviour

Results indicated that more computer use including using the internet and gaming, may lead to poorer motor inhibitory skills. However, direction of the finding and causality are unknown. It may also be that poor inhibitory control leads to more computer use as was found in a study of Little and colleagues,[47] who reported that diminished inhibitory control might underlie excessive computer use and even game addictions. Either way, our findings are relevant to health issues, because of the worldwide increase of sedentary behaviour among youth[4] and the importance of inhibitory control for daily life.[48]

### Limitations and future research

One limitation is that measures of physical activity and sedentary behaviour were based on self-report. While we acknowledge the limitations of this method, self-report measures have been shown to provide valid measures of time spent in sedentary behaviour [12], and unlike other measures (such as pedometers or accelerometers) are able to provide insight into the types of activities (e.g., gaming, doing homework or watching TV[49]). For future research, it is recommended to use objective measurements next to self-reports, such as accelerometers to objectify measurements of physical activities and sedentary behaviour.[50] In addition, as technology and the daily use of new devices grow rapidly, there is a high need for validated but up-to-date questionnaires for measuring sedentary behaviour in children. For instance, the questionnaire we used was developed in 2007, in a time in where tablets, smartphones or online shopping nearly existed. Second, the cross-sectional design used in the present study prevents us from drawing conclusions about the causality underlying the findings. Future research should focus on high-quality RCT’s to draw conclusions about causality and the optimal duration, frequency and intensity of physical activity in preadolescent children to enhance neurocognitive functioning. More specifically, there is no consensus in the literature on beneficial effects of chronic exercise, MVPA and/or cardiovascular fitness in children on neurocognition and executive functioning in particular [4,5], which is complicated by the use of a large variety of tasks including tasks partly appealing to executive functions. Therefore, thoroughly designed studies are required to draw firm conclusions about the effects of exercise in youth. Last, as the present study only included boys, our results may not generalize to girls.[51, 52] In addition, many studies have shown that girls become increasingly less active during adolescence, which in turn has negative effects on health.[53,54]

### Conclusion

The present research complements and extends previous research on benefits of physical activity, and costs of sedentary behaviour, on neurocognitive functioning. The gains of physical activity include key aspects of cognition that are likely to be relevant in any situation that requires basic levels of short-term memory, working memory, and attention (e.g., traffic, conversation, group tasks). The costs of sedentary behaviour seem to include inhibition. Scientifically, the findings may give direction to experimental research needed to unravel cause and effect. Societally, although only part of the puzzle between physical activity, sedentary behaviour, and neurocognition is addressed, we recommend interventions to promote of physical activity and outdoor play to enhance both health *and* neurocognitive functioning in future generations.
